# Zoonotic parasites of dromedary camels: so important, so ignored

**DOI:** 10.1186/s13071-019-3863-3

**Published:** 2019-12-27

**Authors:** Alireza Sazmand, Anja Joachim, Domenico Otranto

**Affiliations:** 1 Department of Pathobiology, Faculty of Veterinary Science, Bu-Ali Sina University, Hamedan, 6517658978 Iran; 2 Institute of Parasitology, Department of Pathobiology, University of Veterinary Medicine Vienna, Veterinaerplatz 1, 1210 Vienna, Austria; 3 Department of Veterinary Medicine, University of Bari, Str. prov. per Casamassima km 3, 70010 Valenzano, Bari, Italy

**Keywords:** *Camelus dromedarius*, Zoonoses, One-Health

## Abstract

With a global population of about 35 million in 47 countries, dromedary camels play a crucial role in the economy of many marginal, desert areas of the world where they survive under harsh conditions. Nonetheless, there is scarce knowledge regarding camelsʼ parasite fauna which can reduce their milk and meat productions. In addition, only scattered information is available about zoonotic parasites transmitted to humans *via* contamination (e.g. *Cryptosporidium* spp., *Giardia duodenalis*, *Balantidium coli*, *Blastocystis* spp. and *Enterocytozoon bieneusi*), as foodborne infections (e.g. *Toxoplasma gondii*, *Trichinella* spp. and *Linguatula serrata*) or by arthropod vectors (*Trypanosoma* spp.). Herein, we draw attention of the scientific community and health policy-making organizations to the role camels play in the epidemiology of parasitic zoonotic diseases also in the view of an increase in their farming in desert areas worldwide.
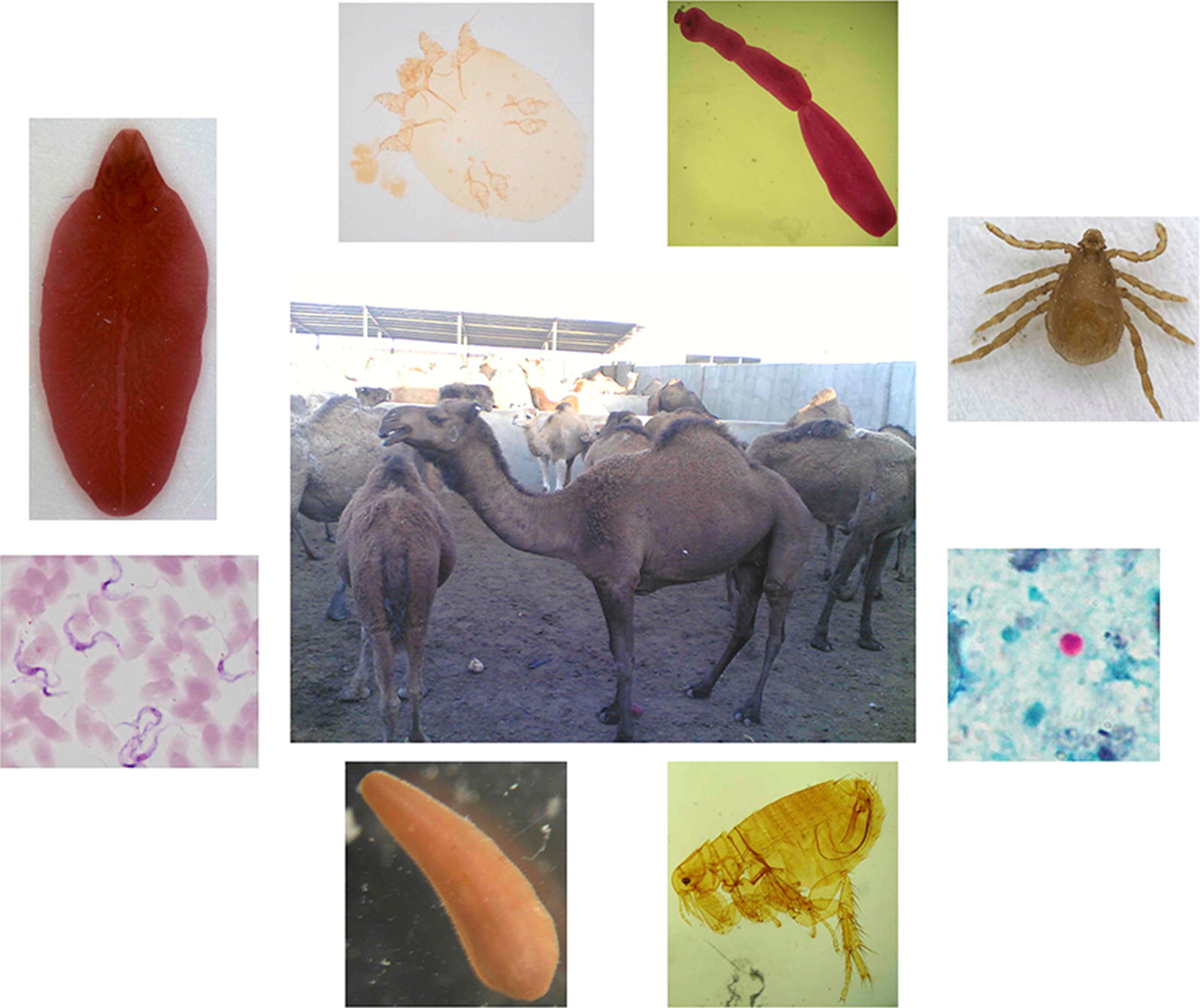

## Background

With a worldwide population of about 35 million, camels are an important source of meat and milk in many regions of the world, mainly in Africa and Asia [1]. The one-humped camel, also known as dromedary (*Camelus dromedarius*), account for approximately 95% of the whole population of Old World Camels and are distributed in 47 countries [1], playing a crucial role in their economy. Therefore, as they are important food sources in semi-arid and arid zones the picture of dromedaries transformed from “ship of the desert” to a “food security livestock” species. The total population of the Old World Camels (OWCs) increased by about 82% from 19 million in 1997 to 35 million in 2017 [1], and the camel industry is in transition from nomadism to intensive production. Although this trend recognizes the economic value of this livestock species as a food source, it could also make camels an increasingly important source for zoonotic disease transmission to humans, especially in resource-poor communities with improper sanitation and medical access. This article reviews the current knowledge on zoonotic parasites reported from camels and gaps on the topic that should be addressed in future research.

## What are camels and why they are important?

The word ‘camel’ refers to any of the members of the family Camelidae including Old World Camels (OWC) and New World Camels (syn. South American camelids, NWC) [2]. The origin of the OWC traces back to around 40 million years ago when the first ancestors of the camelid family were found in North America before migrating *via* the Bering Land Bridge to the eastern hemisphere (the “Old World”) [3] (Table 1). Compared with other animal species (e.g. dogs), the domestication of the dromedary camel took place rather late in human history, approximately 3000 years ago [4]. The dromedary camel is specifically adapted to life in hot, arid areas of the world, notably the Middle East, Africa and India, with a considerable feral population in Australia [5]. Unique physiological peculiarities of dromedaries in circulatory system, respiratory system, water economy mechanism, heat tolerance, etc. enable them to survive almost one week with little or no food and water [6], making them suitable also for trade and trafficking over longer distances in arid areas. Indeed, they are utilized since ancient times for transportation of people, goods, warfare and as draft animals including in agriculture and in local industry. Furthermore, they provide food (meat and dairy products) with great nutritional value, wool and leather in regions of the globe where the common ruminant livestock species (cattle, sheep and goat) cannot be used for this purpose. In the year 2017, camels produced 2,852,213 tons of milk and 630,210 tons of meat [1]. In this article “camel” refers only to “dromedary camel”.

**Table 1:** Taxonomic classification of camelids and other artiodactylids [2]

Taxonomic rank	Scientific name	Common name
Order	Cetartiodactyla	
Suborder	Tylopoda	
Family	Camelidae	
Subfamily	Camelini (Old World camelids)	
Genus	*Camelus*	
Species	*Camelus dromedarius*	Dromedary camel
	*Camelus bactrianus*	Bactrian camel
	*Camelus bactrianus ferus*	Wild Bactrian camel
Subfamily	Lamini (New World camelids)	
Genus	*Lama*	
Species	*Lama glama*	Llama
	*Lama guanacoe*	Guanaco
Genus	*Vicugna*	
Species	*Vicugna pacos*	Alpaca
	*Vicugna vicugna*	Vicuña
Subspecies	*V. vicugna mensalis*	Peruvian vicuña
	*V. vicugna vicugna*	Argentinean vicuña

## Zoonoses parasites of camels

About 65% of the articles on zoonotic pathogens of camels published between 1970 and 2018 focused on Middle East respiratory syndrome (MERS), hydatidosis, brucellosis and Rift Valley fever [7]. Camel echinococcosis is the most studied zoonotic parasitic infection affecting humans but *Toxoplasma gondii*, *Cryptosporidium* spp., *Fasciola* spp., *Trichinella* spp. and *Linguatula serrata* originating from camels are also considered as major public health risks [7]. Relatively few parasites of camels are specific for this host species [8], whereas many others that infect camels are (i) non-zoonotic but with a large host range; or (ii) of zoonotic concern. Transmission of zoonotic parasites includes different routes of infection such as faecal contamination (e.g. *Cryptosporidium* spp., *Giardia duodenalis*, *Balantidium coli*, *Blastocystis* spp., *Enterocytozoon* spp.), or consumption of raw or undercooked infected tissues and milk (e.g. *Toxoplasma gondii*, *Trichinella* spp., *Linguatula serrata*).

In addition, camels serve as reservoir hosts for *Trypanosoma evansi*, or may be infected by gastropod-borne trematodes (e.g. *Fasciola* spp., *Dicrocoelium dendriticum* and *Schistosoma* spp.) or metacestode larvae of zoonotic tapeworms, such as *Echinococcus granulosus* (*s.l*.). Moreover, camels are a blood source for several haematophagous ectoparasites, such as ticks and fleas, which ultimately may transmit zoonotic viral and bacterial pathogens (e.g. Crimean-Congo hemorrhagic fever virus, *Coxiella burnetii*, *Anaplasma* spp.,  *Rickettsia* spp., *Bartonella* spp. and *Yersinia pestis*) [9]. These parasites and infections have been detected in camels in Asia and Africa while there is not much known about the parasite fauna of camels in Australia (see section “Parasites of camels in Australia” below). The current taxonomic status of the zoonotic parasites discussed in this article is shown in Table 2.

**Table 2:** Taxonomic status of major zoonotic parasites of camels discussed in this article as classified by Ruggiero et al. [10]

Kingdom	Phylum	Class	Order	Family	Genus
Protozoa	Euglenozoa	Kinetoplastea	Trypanosomatida	Trypanosomatidae	*Trypanosoma*
Protozoa	Metamonada	Eopharyngia	Diplomonadida	Giardiidae	*Giardia*
Protozoa	Microsporidia	Minisporea (= Microsporea)	Minisporida (= Minisporea)	Enterocytozoonidae	*Enterocytozoon*
Chromista	Ciliophora	Litostomatea	Vestibuliferida	Balantidiidae	*Balantidium*
Chromista	Miozoa	Coccidiomorphea	Eimeriida	Sarcocystidae	*Toxoplasma*
Chromista	Miozoa	Gregarinomorphea	Cryptogregarida	Cryptosporidiidae	*Cryptosporidium*
Chromista	Bigyra	Blastocystea	Blastocystida	Blastocystidae	*Blastocystis*
Animalia	Platyhelminthes	Trematoda^a^	Plagiorchiida	Fasciolidae	*Fasciola*
Animalia	Platyhelminthes	Trematoda^a^	Diplostomida	Schistosomatidae	*Schistosoma*
Animalia	Platyhelminthes	Cestoda^a^	Cyclophyllidea	Taeniidae	*Echinococcus*
Animalia	Nematoda	Dorylaimea	Trichocephalida	Trichinellidae	*Trichinella*
Animalia	Arthropoda	Arachnida	Sarcoptiformes	Sarcoptidae	*Sarcoptes*
Animalia	Arthropoda	Maxillopoda	Porocephalida	Linguatulidae	*Linguatula*

^a^Neoophora *sensu* Ruggiero et al. [10]

### Protozoan parasites in camel faeces

A wide range of gastrointestinal protozoan parasites develop exclusively in camels [11]. Although scientific data are available about infections of camels with several species of parasites of zoonotic importance (e.g. *Cryptosporidium* spp., *Giardia duodenalis*, *Blastocystis* spp., *B. coli*, *Enterocytozoon bieneusi*) their impact on human health has not been confirmed in *ad-hoc* studies except only one documentation on zoonosis linked with camels and that is from Iran [12]. Undoubtedly, the most investigated gastrointestinal protozoan genus in camels is *Cryptosporidium* [13]. Cryptosporidiosis is one of the major zoonotic parasites associated with food-borne and water-borne outbreaks [14]. Of the 39 valid species and approximately 70 host-adapted *Cryptosporidium* genotypes (which do not yet have species names), over 20 have been identified in human patients causing asymptomatic or mild to severe gastrointestinal disease [15]. So far, *C. parvum* subtype IIaA17G2R1 (a common zoonotic subtype reported in humans and animals worldwide), *C. parvum* genetically related to the *C. hominis* If subtype family, *C. andersoni*, *Cryptosporidium* rat genotype IV and a novel genotype (named “camel genotype”) have been confirmed in dromedary camels by PCR and sequencing [13, 16–18]. There is only one documentation of zoonosis linked with camels from Iran where 24 of 100 people in long-term contact with camels were found infected with *Cryptosporidium* spp. [12]. Although *C. parvum* and *C. andersoni* identified in camels are potentially infectious for humans, no confirmed direct association between camels and human infections have been reported, in contrast to other livestock such as cattle [19].

*Giardia* infection is extremely common in both industrialised nations and developing countries, and is responsible for about 280 million human cases of diarrhoea every year [20]. Currently eight species with over 40 animal host species have been reported, of which only *Giardia duodenalis* infects humans [20]. There is only one report on microscopical diagnosis of *Giardia* cysts and trophozoites in dromedary camels [21] with no molecular-based study on the species and genotypes. *Giardia duodenalis* assemblages A and B are the predominant assemblages in humans, but assemblage E is increasingly reported from human patients and assemblages C, D and F have occasionally been identified from human patients [22]. It is predictable that camels, like other hoofed animals, are primarily infected with the zoonotic assemblage E, but the occurrence of other assemblages could clarify the zoonotic potential of camel giardiosis.

Balantidiosis caused by *B. coli* is a zoonotic disease and pigs, non-human primates and humans are known as primary reservoirs [23]. Indeed, human populations living in close proximity to domestic pigs are naturally resistant and mostly without any clinical manifestation, though a case fatality rate of 30% has been reported in acute balantidiosis with intestinal perforation or fulminating haemorrhagic dysentery and shock [23]. In absence of pig raising, such as in some Middle-Eastern countries, camels play a major epidemiological role in the transmission of *B. coli* [24]. Because of the pleomorphism of balantidial trophozoites and the host range, taxonomy of this genus is controversial. However, as for other mammalian hosts [25] balantidia from camels, previously named *Balantidium cameli* [26], are now referred to as *B. coli*, a species causing widespread infection with infection rates of up to 23% [27]. Recent studies on the genetic diversity of *Balantidium* spp. and *Balantidium*-like cyst-forming ciliates, such as *Buxtonella*, suggest that genetic analyses are needed to explain the real spectrum of intestinal ciliates as the cysts are morphologically indistinguishable. *Buxtonella sulcata*, another ciliate with a worldwide distribution, is mainly found in the cecum of cattle but also of camels [28]. Finding of *Buxtonella*-like ciliates in primates opened the hypothesis that *Buxtonella* may also be a pathogen in humans [29], and the possible transmission from camels to humans should be further investigated.

The genus *Blastocystis* comprises at least 17 different ribosomal lineages or subtypes (ST1-ST17), which are arguably separate species [30]. These parasites are estimated to colonize between 1 and 2 billion people globally, with prevalence rates ranging from 5–15% to 50–100% in developed and developing countries, respectively [30, 31]. Humans become infected with ST1 to ST9; however, over 90% of reports are associated with ST1–ST4 [31]. Infections of camels with *Blastocystis* spp. have been reported from Australia [32], Libya [33] and Egypt [34] where ST1, ST3, ST5, ST10, ST14, ST15 or a mixture of them were identified. Interestingly, camel is the host infected with the widest range of STs out of 53 species examined [33]. Three subtypes found in camels (i.e. ST1, ST3 and ST5) can infect humans, suggesting their potential role in transmitting zoonotic subtypes.

Microsporidia are diverse emerging opportunistic pathogens with 200 genera and 1500 species, 17 of which infect humans [35]. Of these, *Enterocytozoon bieneusi*, a ubiquitous protozoan that infects the gastrointestinal tract of a large number of mammals, is frequently recognized in humans [36]. Currently 474 distinctive *E. bieneusi* genotypes from 11 groups have been differentiated out of which Group 1 members are mainly identified in humans whereas others have been suspected [35]. In the only study on dromedaries, Group 6 genotype “Macaque1” and a novel genotype named “Camel-2” (related to members of the group 8 *E. bieneusi* genotypes “Macaque1”, “KB5” and “Horse2”) were identified [16]. The zoonotic potential of *E. bieneusi* genotypes from camels, their frequency and distribution still need to be investigated.

### *Toxoplasma gondii* in camels: what do we know?

Due to its exceptionally wide range of warm- and cold-blooded hosts, *T. gondii* is one of the most successful zoonotic parasites on earth [37]. Indeed, approximately 30% of the world’s human population are infected with this cosmopolitan food- and water-borne parasite [38]. In the USA alone toxoplasmosis accounts for 32,700 disability-adjusted life years (DALYs) annually, being also responsible for 8% of food-borne-illnesses hospitalizations with 86,700 confirmed patients and 330 deaths [39, 40]. Like other livestock, camels acquire *T. gondii* infections through ingestion of sporulated oocysts shed by cats or wild felids in the environment [41]. Antibodies against *T. gondii* in sera of dromedaries from different countries have been determined using various techniques, reporting seroprevalences as high as 67% [42, 43]. It has been estimated that 36% of camels in Africa have anti-*T. gondii* antibodies [44]. Moreover, *T. gondii* DNA has been detected in the blood of Iranian dromedaries [45]. Clinical and congenital toxoplasmosis, however, are limited to a few reports and probably underestimated in camels [46–49]. *Toxoplasma gondii* cysts have been isolated from camel meat [43] but predilection sites of *Toxoplasma* cysts have not been comprehensively investigated in this host species. The rooted habits of nomadic populations of some African and Asian communities of raw camel liver consumption [43, 50] suggest that this could represent a risk factor for infection of humans, as *T. gondii* is frequently isolated from the livers of domestic ruminants and horses [51]. In addition, consumption of camel milk is becoming increasingly popular in recent years because it is richer in vitamin C and iron than cow’s milk, with important therapeutic effects for the treatment of type 1 diabetes and reduction of allergies in children [52]. The implication of unpasteurized camel milk as a source of human toxoplasmosis [53] suggests that consuming raw milk or dairy products without pasteurization or heat treatment (e.g. Shubat) could be a risk for human health. Little is known about the genetic characteristics of *T. gondii* genotypes infecting camels. Some surveys showed the occurrence of all three conventionally defined clonal lineages (Types I, II and III) in camel meat and milk [18, 54, 55]. All of these types have also been isolated from human patients [56]. Since the conventional nomenclature of *Toxoplasma* isolates does not sufficiently delineate the plethora of existing genotypes [57], multilocus PCR-RFLP genotyping should be applied to improve current understanding of the transmission dynamics of infected camels to people consuming their meat and dairy products.

### *Trypanosoma evansi* in camels

Camels are affected by several *Trypanosoma* species [58]. While *T. evansi*, the etiologic agent of “Surra” is the more prevalent trypanosome species of camels [59], *T. brucei*, *T. congolense* and *T. vivax* are found at low infection rates [60, 61]. Due to a partial loss of *T. evansi* mitochondrial DNA, which occurred during its segregation from *T. brucei* [62], this species can be mechanically transmitted by virtually all biting flies, so its geographical distribution is potentially unlimited. *Trypanosoma evansi* affects a wide range of domestic and wild mammals in Africa, Asia and South America [63], and recent outbreaks of infection amongst dromedary populations on the Canary Islands, in mainland Spain and France demonstrated the potential of the parasite to spread rapidly even in non-endemic areas [64]. In dromedaries, the infection may cause significant morbidity and great impairment of productivity and mortality [65]. It is assumed that the spread of *T. evansi* among camels with the consequence of fatal anaemia weakened the Arab-African Muslim forces in their prolonged battle against Christendom, as they relied heavily on camels and equids for transport and economy [66]. *Trypanosoma evansi* has its highest prevalence in camels compared to other animal hosts such as buffaloes, cattle, dogs, equids and small ruminants [63], but in contrast to other livestock species, in camels the economic burden of this infection has not been evaluated [67]. Human cases of *T. evansi* infection have been reported from India, Sri Lanka, Egypt and Thailand [68–70]. For a decade it was hypothesized that human susceptibility to *T. evansi* could be linked to insufficient or missing levels of human trypanocide apolipoprotein L1 (APOL1), a trypanocidal component of normal human serum [71]. However, a recent report of infection in a patient with no previous immunological risk, 2 wild-type APOL1 alleles and a normal serum APOL1 concentration suggested that *T. evansi* is a true zoonosis with a risk of infection for the general population [70].

Traditionally tabanids, muscids and hippoboscids are considered to be mechanical vectors of *T. evansi*, and several species of the genera *Ancala*, *Atylotus*, *Chrysops*, *Haematobia*, *Haematopota*, *Hippobosca*, *Pangonia*, *Philoliche*, *Stomoxys* and *Tabanus* have been collected directly from camels or their surroundings [9, 72–74]. *Stomoxys calcitrans*, *Stomoxys niger*, *Tabanus taeniola*, *Tabanus par* and *Tabanus subangustus* collected on cattle have been identified to be infected with *T. evansi* [75]. However, there is no molecular confirmation on the role of definite fly species as vectors of *T. evansi* in camels.

### Hydatidosis in camels

Cystic echinococcosis (CE) is a major zoonotic infection with worldwide distribution caused by the larvae of the tapeworm *Echinococcus granulosus* (*s.l*.). CE causes considerable medical costs and economic losses in endemic areas. Camels are intermediate hosts for several zoonotic *Echinococcus* species, being important in their epidemiology [76]. Cysts are commonly found in the lungs and, to a lesser extent, the liver of camels, resulting in carcass condemnation and, subsequently, great economic losses. In Iran where CE is endemic, the annual monetary burden of CE has been estimated at 232.3 million USD, out of which the loss due to condemnation of infected camel livers amounts to approximately 600,000 USD [77]. Infection of camels with cysts of *E. granulosus* (*s.s.*), *E. ortleppi* and *E. canadensis* (formerly G1, G2, G3, G5, G6) have been reported [78–80], all of them being causative agents of human CE [80]. The prevalence of *Echinococcus* species infecting dromedaries differs in various studies, i.e. in studies from Iran and Ethiopia the most prevalent species isolated from camels was *E. granulosus* [81, 82], while in Nigeria and Oman most of the isolates were identified as *E. canadensis* [83, 84]. A comprehensive review of all the available information about *Echinococcus* species infecting dromedaries is needed to show which species occur more commonly in each region, continent and at global scale.

### Linguatulosis in camels

Humans become infected with the cosmopolitan pentastomid *Linguatula serrata* by ingestion of eggs from the faeces of infected dogs or consumption of raw or undercooked infected viscera of intermediate ruminant hosts and camels [85]. Human nasopharyngeal infection by the so-called tongue-worm can affect the nasopharynx, throat, eyes, lymph nodes, nose, oral cavity, lungs and liver [85]. Reliable data on the rate and geographical range of the infection in dogs are unavailable and diagnosis is challenging as infections in dogs are often asymptomatic [86]. However, clinical linguatulosis is increasingly reported in pet and stray dogs worldwide [87] and zoonotic cases of *L. serrata* infection are recorded from several countries in Asia, Europe, Africa and the Americas [85]. In camels, infection with *L. serrata* larvae in the liver, lungs, spleen, mesenteric and mediastinal lymph nodes have been reported from Iran, Egypt and Sudan [79, 85]. Although consumption of improperly cooked and raw liver of infected intermediate hosts are major sources of zoonotic infection, the actual sources of infection in human patients have not been documented sufficiently, and camels may play an important role in the epidemiology of human and canine linguatulosis.

### Trichinellosis in camels

The genus *Trichinella* comprises nine species and three genotypes occurring in birds, reptiles and more than 150 domestic and wild mammalian species [88]. Some species within the genus cause a meat-borne zoonosis responsible for 5751 cases and five deaths per year [88]. Although human risk for trichinellosis has historically been linked to *Trichinella spiralis* acquired from domestic pig or wild boar, meat of other omnivorous or carnivorous animals, but also from herbivorous domestic livestock and horses, have been implied in the occurrence of human trichinellosis [89]. *Camelus* sp. was listed as host of *T. spiralis* in India in 1977 [90], and as a sequel to a severe outbreak of trichinellosis in Germany attributed to spiced and dried camel meat, illegally imported from Cairo, a few confirming studies were conducted [91]. While, the camel origin of the exotic dish incriminated for that outbreak could not been confirmed, experimental infection of camels with *T. spiralis* from pork meat resulted in a high *Trichinella* burden of the smooth and striated muscles [92]. Based on these finding and on the fact that meat from sheep, cattle and horse has been recognized as source of human trichinellosis in several outbreaks [89], the role of camels in the epidemiology of trichinellosis needs further investigation. This should be a priority, also considering that eating raw camel meat is popular among camel nomads in some regions, and severe foodborne outbreaks of plague have occurred due to this habit [93].

### Zoonotic gastropod-borne trematodes in camels

Diseases caused by gastropod-borne helminths are estimated to affect more than 300 million people worldwide [94]. Camels can potentially play a role in the maintenance and transmission of several gastropod-borne trematodes in areas where both parasites and hosts are present. Fasciolosis is a food- and water-borne disease caused by *Fasciola hepatica* and *Fasciola gigantica* liver flukes. Human fascioliasis is an important public health problem and is considered a highly neglected tropical disease with estimated 35 to 72 million people infected worldwide [95]. Infections of camels with both fluke species with prevalences of up to 15% have been recorded [79, 96]. Meanwhile, reports of human infections with *D. dendriticum* flukes are increasing, mainly due to the expansion of arid areas and the increase in anthelmintic resistance [97]. Dicrocoeliosis in humans is poorly known and probably underestimated; however, infections have been reported from several countries [98] and many animal species, including camels, have been demonstrated to harbor fertile adult flukes and excrete eggs with their faeces [99, 100].

Schistosomiasis is an infectious disease that affects more than 230 million people worldwide [101]. Four *Schistosoma* species, *Schistosoma bovis*, *Schistosoma mattheei*, *Schistosoma indicum* and *Schistosoma turkestanica* (syn. *Orientobilharzia turkestanicum*, *Ornithobilharzia turkestanicum*), have been reported in camels [102–104]. *Schistosoma bovis* and *S. mattheei* have also been described in humans [105], as well as human cercarial dermatitis caused by *S. turkestanica* has been reported [106].

### Arthropods infesting camels

Camels can be infested by a wide range of external parasites that irritate, injure or debilitate them [9]. Moreover, different ticks and flies are biological and mechanical vectors of several viruses, bacteria and parasites that can induce human infections [9]. Camel mange caused by *Sarcoptes scabiei* var. *cameli* is a major threat to camel health and production as it is extremely contagious [9]. It is considered second only to Surra in terms of losses in camels [107] and its transmission to humans, particularly camel attendants and riders, is well-known since ancient times [108]. Infestation of camels with ticks of the genera *Rhipicephalus*, *Hyalomma*, *Dermacentor*, *Ixodes*, *Amblyomma*, *Argas*, *Otobius* and *Ornithodoros* is often reported [9]. Almost all of the tick genera above encompass species of known or putative vectors for zoonotic pathogens [109]. For instance, viruses with zoonotic potential have been detected by molecular methods in ticks collected from camels, including Crimean-Congo hemorrhagic fever virus [110], Alkhurma hemorrhagic fever [111], Dhori virus and Sindbis virus [112], Kadam virus [113] and Toghoto virus [114]. In addition, some bacteria of public health importance such as *Coxiella burnetii* [115], *Rickettsia* spp. (*R. aeschlimannii*, *R. africae*, *R. sibirica mongolitimonae*), *Bartonella* (*B. bovis* and *B. rochalimae*), *Anaplasma phagocytophilum* and *Borrelia burgdorferi* (*s.l*.) [116] have been detected in the blood of camels and/or ticks parasitizing them.

As *Yersinia pestis* has been isolated from *Xenopsylla cheopis* rat fleas captured near camel corrals [50] it has been assumed that they may act as vectors for plague in camels, which in turn, can infect humans directly or carry infected fleas close to humans. Infection of camels with plague has been suspected for a long time [117], and the role of this animal species in outbreaks in different countries have been documented although infection might show no overt symptoms [118]. Transmission of plague form camels to humans has been reported in Kazakhstan, where from 1907 to 2001, human plague was acquired from camels in 400 instances [119].

## Parasites of camels in Australia

There is controversy about the estimated population of feral dromedary camels in Australia with an estimated number of between 300,000–1,200,000 camels [5, 120]. However, in-depth information about diseases of camels in Australia is scarce. In the only study available [121], cystic echinococcosis was reported with zero cases in 4915 camels examined during meat inspection in abattoirs. According to the current knowledge, the most common parasites of camels in Australia are *Sarcoptes scabiei*, *B. coli* and camel nasal bot fly, *Cephalopina titillator*, whereas *Trichuris tenuis*, *Camelostrongylus mentulatus*, *Cooperia pectinata*, *Nematodirella dromedarii*, *Haemonchus* sp., *Trichostrongylus* sp., *Cooperia* sp., *Nematodirus* sp., *Nematodirella* sp. and *Eimeria cameli* are less common. *Cryptosporidium parvum* was reported from a dromedary calf. Tapeworm infections and taeniid cysts were absent. *Trypanosoma evansi* was imported into Western Australia in 1907 with camels but was diagnosed and quickly eradicated before spreading [13, 67, 122–124]. Of the parasites listed, *S. scabiei*, *C. parvum* and *B. coli* are zoonotic.

In recent years, export of live camels from Australia to the Middle East has increased [125]. Apart from animal welfare issues, and possible intolerance of Australian camels to the climate of the Persian Gulf, the trade of animals on a global scale has implications for parasite spread. Recently it was suggested that treatment of internal parasites in livestock in the country of origin may help in preventing entrance of helminths to barns, flocks and pastures in the country of destination [126]. Since not much is known about the parasite fauna of Australian camels it is conceivable that certain parasite species are transferred to areas of the Middle East where they are currently absent. Conversely, the susceptibility of feral camels from Australia to the diverse parasite fauna of the Middle East upon their arrival remains to be discussed.

## Conclusions

Due to their increasing importance as a livestock animal in marginal, desert areas of developing countries, the role of camels in the epidemiology of zoonotic parasitic infections needs to be further investigated, especially in view of the risk factors associated with them. So far, research on parasites of camels has focused on case reports or prevalence surveys by microscopical examination of faecal samples or blood smears [21, 127, 128], whereas identification of parasites by molecular tools and phylogenetic analyses are scarce. Considering this, it would be important to perform molecular investigations on parasites of camels and of people having direct contact to them, in order to improve the current understanding of transmission dynamics in epidemiological studies. In addition, the role of camels as hosts for zoonotic pathogens such as *Trichinella* needs confirmatory evidence, and further studies of infectivity, pathogenicity, muscle larvae distribution and antibody development are necessary to understand the role of camels in the maintenance, distribution and transmission of this parasite. As a large portion of the camel population is kept in communities lacking equipment and trained personnel for carrying out parasitological examinations, there is a need for the development of rapid diagnostic tests for the detection of the most important camel parasites. Moreover, international and local organizations must work to increase the awareness of the zoonotic risk of camel parasites and the ways of pathogen transmission for people working in close contact with camels. Most importantly, the high risk of acquiring zoonotic infections by consumption of raw milk, meat and liver of infected camels, as well as their role in maintaining zoonotic transmission of hydatidosis must be communicated in the best possible way.

## Data Availability

All data generated or analyzed during this study are included in this published article.

## Abbreviations

OWC: old world camelids
NWC: new world camelids
MERS: Middle East respiratory syndrome
PCR: polymerase chain reaction
DALY: disability-adjusted life year
APOL1: apolipoprotein L1
CE: cystic echinococcosis
